# Survival Difference in Advanced-Stage Cervical and Ovarian Cancer Patients Treated with Concomitant Modulated Electro-Hyperthermia in Comparison to Classic Treatment Modalities: Results of a Pilot Study and Meta-Analysis

**DOI:** 10.3390/medsci14010105

**Published:** 2026-02-22

**Authors:** Ivan Panczel, Magdolna Herold, Erika Borbenyi, Daniel Horanyi, Zoltan Novak, Magdolna Dank, Attila Marcell Szasz, Zoltan Herold

**Affiliations:** 1 Division of Oncology, Department of Internal Medicine and Oncology, Semmelweis University, H-1083 Budapest, Hungary; 2 National Institute of Oncology, H-1122 Budapest, Hungary; 3 Department of Clinical Oncology, Semmelweis University, H-1122 Budapest, Hungary

**Keywords:** cervical cancer, concomitant therapy, modulated electro-hyperthermia, ovarian cancer, meta-analysis

## Abstract

Background: Modulated electro-hyperthermia (mEHT) is one of the latest advancements in the field of oncological hyperthermia. Previous studies investigating mEHT revealed that it is safe and effective; however, no meta-analysis was conducted either in cervical or ovarian cancer. Methods: A single-institute pilot case series and a meta-analysis were conducted. Advanced stage cervical and ovarian cancer cases were included. In the pilot study, mEHT treatments were conducted using the Oncotherm EHY-2000+ and the EHY-2030 devices with 2–3 treatment sessions per week. Results: For the meta-analysis, a total of five studies were identified, with 160 and 31 cervical and ovarian cancer patients, respectively. In addition, 175 standard-of-care-treated cervical cancer patients were also identified as controls. The 1- and 2-year survival rate of the cervical cancer patients treated with mEHT was 87.61% [95% confidence interval (CI): 71.31–100%] and 78.13% (95% CI: 53.02–100%). Compared to the controls, the 2-year survival rates (78.13% vs. 58.86%) were significantly better in the mEHT-treated cohorts (odds ratio: 0.4143, *p* = 0.0441; hazard rate: 0.6607, *p* = 0.0103). The 1- and 2-year survival rates of ovarian cancer patients were 45.46% (95% CI: 5.97–84.95%) and 32.83% (95% CI: 0–79.57%), respectively. The result of our institutional data strengthened the results of the meta-analysis. Conclusions: Using mEHT, a significantly higher 2-year survival rate can be achieved in cervical cancer. In this setting, a wider testing/application of the modality is warranted. In the case of ovarian tumors, the available knowledge is minimal, and applicability and efficacy studies are urgently needed.

## 1. Introduction

Cervical and ovarian malignancies are one of the most frequent cancers arising in women. In 2022, 986,904 new cases emerged, and more than 555,830 deaths occurred worldwide [1]. Despite the major advancements in treatments, the overall survival and patient outlook for these malignancies is still extremely poor, with a 5-year overall survival of 10–40% [2]. This in part is due to the lack of symptoms and limited diagnostic tools to detect malignancies at early stages. Treatment options in advanced-stage diseases include chemo(radio)therapy and hormonal therapies. Moreover, in recent years, various new treatment options also emerged, including but not limited to immunotherapy, targeted gene therapy and hyperthermic methods [3,4].

In oncology, concomitant hyperthermia is the artificial raising of the temperature of the tumor and its surrounding to 39–42 °C using full-body or regional heating methods [5]. One of the latest advancements in the field is modulated electro-hyperthermia (mEHT). It relies on a capacitive coupled impedance matched method, whereby a 13.56 MHz radiofrequency current is conducted through a given target [6]. This current is driven through a conductor head which is placed above the oncological target. For this reason, it would perhaps be more accurate to classify it as an electrotherapy rather than hyperthermia. Malignant cells possess different biocellular and bioelectric properties to physiological cells, which makes it possible to selectively target only the tumor-infected targets and avoid all other healthy tissues. The electric current not only causes local hyperthermia in the cells, which in turn causes cell apoptosis, but also initiates a series of chain reactions within the cells, leading to the formation of damage-associated molecular patterns (DAMPs), ultimately (re-)activating the immune system [7,8]. mEHT also has an abscopal effect, meaning that, once the local treatment has been completed, a systemic response is activated, creating a tumor vaccine-like effect by activating immune cells, acting on micro- and macro-metastases and creating immune memory [9,10]. mEHT is reported to be a safe, non-invasive option to treat locally disseminated tumors of all variants when conventional methods have become ineffective. It has to be mentioned that, although there are many theoretical, cellular and animal model studies, clinical data is still scarce on the subject. It has been explored in various malignancies, such as breast, pancreatic and brain cancer. However, in most cases, the number of patients included in the studies is (very) low but with a high heterogeneity; there is only a single randomized study, and in many cancers only case reports are available. Generally, this therapy is recommended to be used 1–3 times per week, with each treatment lasting 60 min [4]. A minimum of a 48 h long interval between two treatments must elapse to allow the heat shock protein response to form and naturally exhaust itself; otherwise, the treatment will be ineffective and, in some cases, may even be harmful [4,10,11].

Although all previously published results suggest that the concomitant addition of mEHT to any treatment protocol shows an improvement in the measured variable, to date, its use—not only in gynecological but in oncology in general—is very limited with only minimal literature published on the topic. Therefore, an exploratory meta-analysis was performed to summarize and bring light to all the available literature regarding the use of mEHT in cervical and ovarian tumors and thus provide data on the potential feasibility of this treatment modality. Our further goal in the meta-analysis was to assess the survival advantage of mEHT compared to traditional treatment methods and whether it is worth planning/organizing further, e.g., randomized studies on the topic. Moreover, the further aim of our study was to present the results of cervical and ovarian cancer patients who were treated with concomitant mEHT in our own single-institute observational pilot case series.

## 2. Materials and Methods

The study consisted of two main parts. First, the descriptive data of late line/salvage gynecological cancer patients treated with standard-of-care methods followed by mEHT were reviewed retrospectively. Second, a meta-analysis was conducted. The study was approved by the Regional and Institutional Committee of Science and Research Ethics, Semmelweis University (SE TUKEB 8/2017, SE TUKEB 8-1/2017; approval date of the latest version: 9 January 2023).

### 2.1. Patient Selection and Collection of Clinicopathological Data

Patients attended the Division of Oncology, Department of Internal Medicine and Oncology, Semmelweis University, Budapest, Hungary, between 2017 and 2020. A total of six consecutive patients with advanced-stage gynecological malignancies were included. All patients provided their written informed consent to participate. Two and four of the six patients were treated for cervical and ovarian cancer, respectively. Exclusion criteria included <18 years of age, a previous and/or synchronous other malignancy, active-phase autoimmune disease or an active infection, mental illnesses, and a >1 Eastern Cooperative Oncology Group (ECOG) performance score.

All relevant anamnestic data including ancestorial, gynecological and general anamnesis, age at cancer diagnosis, tumor location and therapy details were obtained. Data regarding the mEHT treatment were the device details, date of first and last treatment, number of treatments received, emitted maximal dose, side effects experienced during therapy and, where applicable, reason for the end of treatment. Overall survival was calculated as the time elapsed between the initiation of the mEHT treatment and the date of death or the last follow-up (31 May 2025). Definition and grade of adverse events were classified based on the Common Terminology Criteria for Adverse Events (CTCAE) v6.0 [12].

### 2.2. Modulated Electro-Hyperthermia

The mEHT treatment was performed in all six included patients after classical treatment modalities had been exhausted. The Oncotherm EHY-2000+ (Oncotherm Kft., Budaörs, Hungary) and the Oncotherm EHY-2030 (Oncotherm Kft., Budaörs, Hungary) devices were used in 3–3 patients, respectively. Both devices were set to 15.56 MHz frequency with modulated amplitudes, capacitive coupling, and using a 30 cm wide applicator (electrode). The 60 min long treatment was performed in supine position placing the electrode above the intended tumorous target area 2–3 times per week until further disease progression. The planned maximal dosage output was 100 W for 30 min in the first week of the treatment, followed by 150 W for 30 min at all later treatment visits. As per the manufacturer’s description, a step-up phase should be applied in the first 30 min of the treatment. During this, the device’s starting power is set to 100 W (during the first week: 50 W), and the power is increased by 5 W every 3–5 min. In cases where any of the patient(s) felt any discomfort, the device’s power was lowered to a tolerable power, paused or finished if the discomfort was intolerable. Checking for sign of subcutaneous adipose burn was performed during the routine imaging studies (ultrasound, magnetic resonance imaging or computed tomography within every 3–6 months).

### 2.3. Search Strategy and Data Extraction for the Meta-Analysis

The meta-analysis was registered in the PROSPERO database (CRD420251161662), and it was conducted following the Preferred Reporting Items for Systematic Reviews and Meta-Analyses (PRISMA) guidelines (Table S1) [13]. Three main databases—BioMed Central (BMC), Cochrane Library, and the PubMed—Medical Literature Analysis and Retrieval System Online (MEDLINE)—were selected to conduct the searches. No filtering, language restriction and/or automation tools were used. Articles published until the 1st of July 2025 from their first inception were selected. The published literature collected by the manufacturer of the mEHT device’s website (https://oncotherm.com/clinical-publications/; accessed on 30 November 2025) was also considered as a source. The search strings used were the following. The term “cervical cancer” or “ovarian cancer” was combined with “modulated electro-hyperthermia”, “electrohyperthermia”, and “modulated hyperthermia” using the logical operator AND. I.P. and Z.H. independently performed the literature search, and any discrepancies were resolved by consensus and, if necessary, by the opinion of M.H. and A.M.S. Inclusion criteria for the studies were the use of any mEHT device to treat gynecological cancer patients in any settings, where relevant survival data was published either in the form of survival curves, survival rates, or the number of deaths observed during specific timeframes. No studies were included where the observation period was short (<1 year), if the data originated from mixed populations (e.g., if treated patients could not be separated from the controls), or if only other metrics than the previously defined survival indicators were available. Reviews, conference abstracts, in vitro and animal studies, and theoretical works were also excluded.

The following data were recorded: the name of the author(s), year of publication, tumor location, study type, type of device used, oncological treatments received, and the 1- and 2-year survival rates. If the 1-/2-year survival rate was not directly presented, but (1) could be read from a corresponding survival curve(s) or (2) calculated manually from the presented data, the 1- and 2-year survival rates were converted from those. In the case of those studies where both mEHT-treated and control patients were also available, hazard rates (HR) and their 95% confidence intervals (CIs) between the two cohorts were also collected.

### 2.4. Statistical Analysis

Statistical analyses were performed within the R version 4.5.2 environment (R Foundation for Statistical Computing, 2025, Vienna, Austria) using the R package meta (version 8.2-1) [14]. Survival rates and HRs were used for the effect size measurement, and random-effects models were performed. As HRs are not normally distributed, their logarithmic value and the corresponding standard error (SE) were used in the meta-models [15]. The heterogeneity variance measure (τ^2^) was estimated using the restricted maximum likelihood and Q profile methods [16,17]. Between-study heterogeneity was tested with Higgins and Thompson’s I^2^ statistic [18]. Publication bias was examined using Begg’s rank correlation test of funnel plot asymmetry [19]. Comparison of mEHT-treated and control cohorts was performed using the Mantel–Haenszel method [16,20,21]. Forest plots were used to graphically present the meta-analysis results.

## 3. Results

### 3.1. Case Series

A total of six gynecological cancer patients were treated with concomitant mEHT at our institution, of which two and four had advanced-stage cervical and ovarian cancer, respectively. All relevant clinical features of the patients, including the anamnestic and mEHT treatment data, can be read in Table 1. At the time of the first mEHT treatment, all patients had an ECOG performance score of 0. The two cervical cancer patients (aged 29 and 57) had late-stage [International Federation of Gynecology and Obstetrics (FIGO) III–IV] cervical cancer at the time of study inclusion. Both cervical cancer patients had previously undergone primary tumor removal surgery, and one of the two patients received chemoradiotherapy (4 cycles of cisplatin + irradiation followed by 16 cycles of taxane + cisplatin + bevacizumab and 5 cycles of bevacizumab monotherapy). In the case where chemotherapy was not introduced, the patient did not need further oncological therapy following the primary tumor removal surgery, and the relapse occurred in the form of FIGO IV-stage carcinosis, where only the best supportive care was optional. The two patients in this cohort received a total of 15 and 18 sessions of mEHT, with an average maximal dose of 138.91 ± 10.73 W. None of the patients showed any signs of adverse effects in this cohort. The mEHT treatment was discontinued in both cases due to the progression of the tumor. Following their first mEHT treatment, the two patients showed an overall survival of 3 and 11 months and a median overall survival of 6.81 months (Table 1).

The four ovarian cancer patients, aged 48, 61, 36 and 88, had FIGO III-IV-stage tumors, and all of them had primary tumor removal with or without cytoreductive surgery. Three of the four patients received adjuvant chemotherapy. In all cases, the chemotherapy entailed a minimum of six cycles of taxane + carboplatin, and one patient received second-line taxane + bevacizumab therapy. The patients in this cohort received a total of 13, 16, 23 and 50 sessions of mEHT. The average maximal treatment output of the device was 128.18 ± 29.44 W. Similar to the cervical cancer patients, the reasons behind stopping the mEHT therapy were disease progression and, in one case, death. Adverse events were reported for two patients in the form of a burning sensation (closest CTCAE 6.0 term: pain of skin; grade: I) and a slight swelling of the inguinal region after a single treatment (CTCAE 6.0 term: localized edema; grade: I). The latter was resolved by the next mEHT treatment without any recurrence or sequelae. The 1-, 2- and 5-year survival rates of the cohort were 100%, 50% and 50%, respectively. Two of the four patients were still alive at the end of the study’s follow-up period. The two deaths occurred on the 13th and 18th month following the first mEHT treatment, and the median overall survival was 17.77 months.

### 3.2. Meta-Analysis

**Figure 1:**
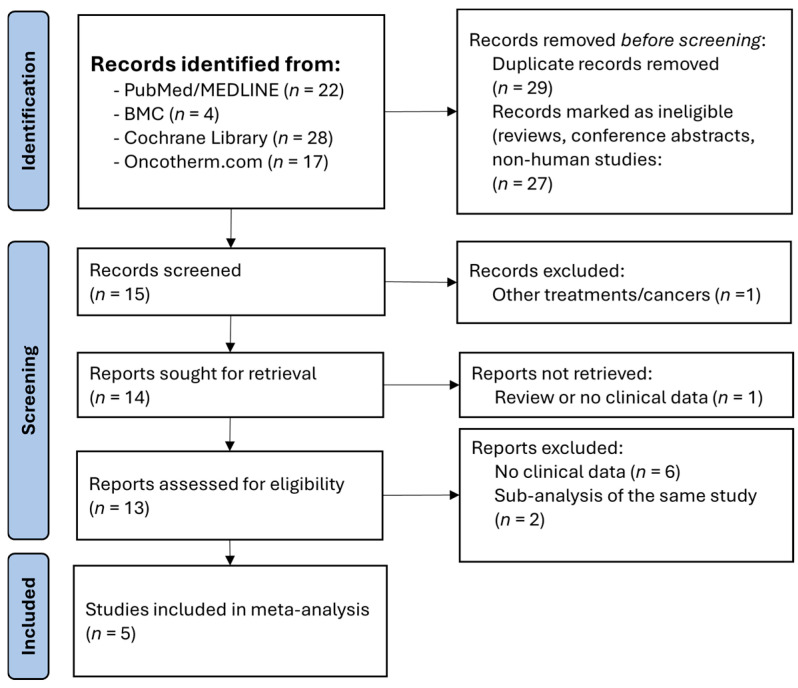
PRISMA flow diagram of studies about modulated electro-hyperthermia in gynecological cancers. BMC: BioMed Central; MEDLINE: Medical Literature Analysis and Retrieval System Online; PRISMA: Preferred Reporting Items for Systematic Reviews and Meta-Analyses.

The electronic database searches for publications regarding the use of mEHT in gynecological cancers resulted in a total of 71 articles. After removing duplicates, experimental and cellular reports, reviews and conference abstracts, 15 articles remained for abstract screening. A further nine articles were excluded due to being review articles, reported precursor information of an ongoing clinical trial, or articles without any data on patient survival. A total of five published studies [22,23,24,25,26] were then considered and accepted for full-text assessment (Figure 1). The two studies of Lee et al. [23,25] were carefully investigated for whether there was an overlapping population. It was found that, in the 2017 study, they investigated mEHT alongside chemotherapy alone [25], while in the 2023 study chemoradiotherapy with and without mEHT was compared [23]. This entailed a total of 366 patients. The data collected was divided into patients with cervical and ovarian cancers for a more precise meta-analysis. Details of the selected five publications can be read in Table 2.

#### 3.2.1. Meta-Analysis of Cervical Cancer Patients

**Figure 2:**
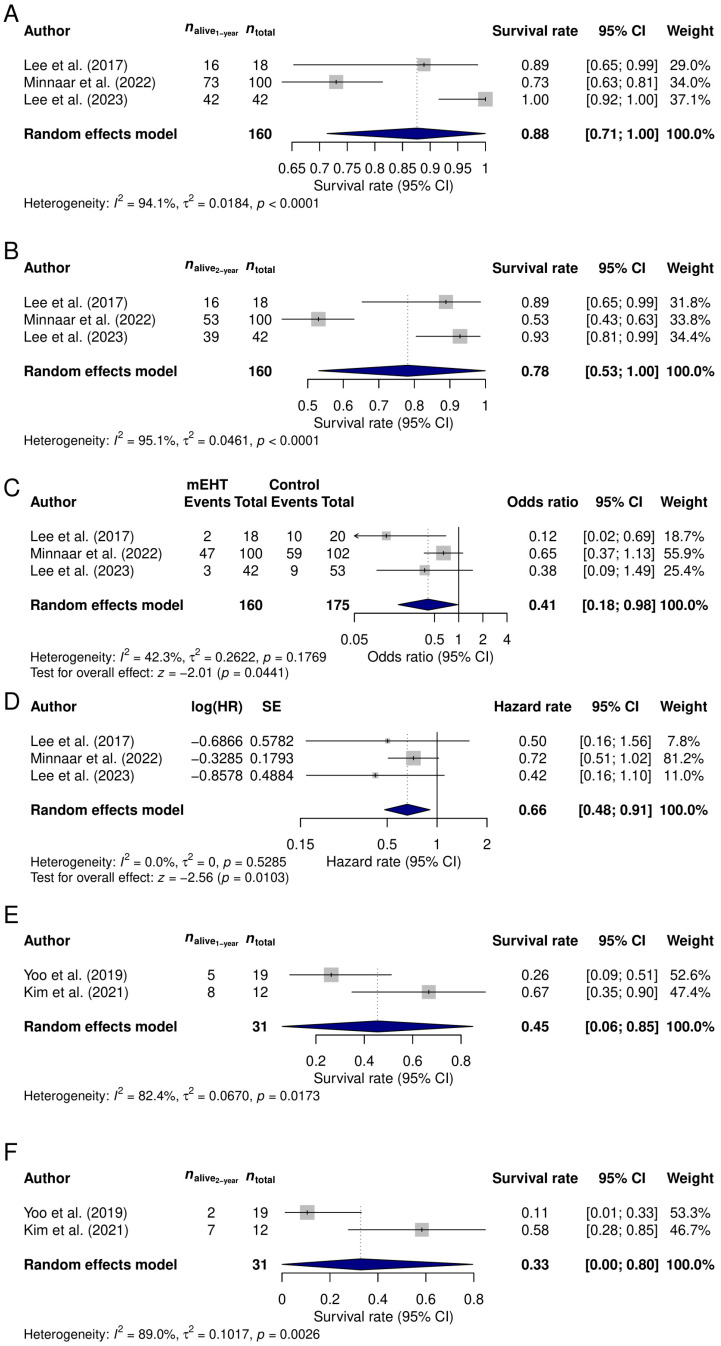
Cervical (**A**–**D**) and ovarian (**E**,**F**) cancer results of the meta-analysis. The 1- (**A**) and 2-year (**B**) survival rates of cervical cancer patients treated with concomitant modulated electro-hyperthermia (mEHT) were 88% and 78%, respectively. Cervical patients treated with concomitant mEHT had better survival whether it was investigated with raw event numbers at 2 years (**C**) or with HRs (**D**) compared to those not receiving the treatment. The 1- (**E**) and 2-year (**F**) survival rates of ovarian cancer patients treated with concomitant mEHT were 45% and 33%, respectively. CI: confidence interval; log(HR): logarithmic value of the hazard rate; *n*: number of patients; SE: standard error; [22,23,24,25,26].

A total of three study results were included in the meta-analysis investigating mEHT in cervical cancer. The 1-year survival rate of the 160 mEHT treated patients was 87.61% (95% CI: 71.31–100%; Figure 2A), while the 2-year survival rate was 78.13% (95% CI: 53.02–100%; Figure 2B). The results of the Begg’s rank tests (*p* = 0.6015 for both models) suggested no publication bias.

All three previous cervical cancer studies compared the mEHT-treated cases (*n* = 160) to patients treated with standard-of-care oncological treatments only (*n* = 175). None of the studies reported any significant differences between the two cohorts, except for the use of mEHT. For this comparison, both the survival rates and HRs were available. The direct comparison of the proportions revealed a 5.56% (87.61% vs. 82.05%; *p* = 0.6843) and a 19.27% (78.13% vs. 58.86%; *p* = 0.2931) 1- and 2-year survival rate difference, respectively. The odds ratios for the death events within the first and second year were also calculated. It was found that, within the first year, the mEHT treatment can somewhat reduce the occurrence of the death events (odds ratio: 0.6584; 95% CI: 0.3723–1.1643; *p* = 0.1508), while the overall survival of the mEHT-treated group at the second year is significantly better (odds ratio: 0.4143; 95% CI: 0.1757–0.9771; *p* = 0.0441; Figure 2C). Similarly to the latter observation, the comparison of HRs showed the same significant difference favoring the mEHT treatment (computed HR between the two cohorts: 0.6607; 95% CI: 0.4813–0.9069; *p* = 0.0103; Figure 2D).

#### 3.2.2. Meta-Analysis of Ovarian Cancer Patients

A total of 31 mEHT-treated ovarian cancer patients were included in the meta-analysis. The 1- and 2-year survival rates of the patients were 45.46% (95% CI: 5.97–84.95%; Figure 2E) and 32.83% (95% CI: 0–79.57%; Figure 2F). Due to the low number of studies included in this analysis, no publication bias could be investigated. Furthermore, the lack of control patients in these studies prevented us from making further comparisons.

## 4. Discussion

To date, gynecological malignancies still pose challenges for clinicians [27]. Their treatment has undergone major changes in recent years, with constantly evolving protocols; however, the classical treatment modalities—surgery, radiotherapy and chemotherapy—remain the mainstay. The former includes, e.g., targeted therapies, immunotherapies, anti-drug conjugates, and personalized treatments based on molecular biomarkers, as well as various hyperthermia methods. The use of hyperthermia has always been a topic of debate in oncology. While some hyperthermic methods have proven to be effective, such as hyperthermic intraperitoneal chemotherapy or radiofrequency ablation, others, like mEHT, are still only available to patients through research studies. Although in some other cancers the use of mEHT is somewhat better detailed [4,5,28], its use in gynecological oncology is less detailed. In this article, we collected the available literature and performed the first and only meta-analysis to date. We found that patients with advanced cervical and ovarian cancers have 88% and 78% and 45% and 33% 1- and 2-year survival rates, respectively. In addition, we performed an explanatory case series from our institutional data as well, which showed somewhat different survival data, possibly related to the “last resort” use of the treatment. Based on the results obtained from the meta-analysis, the concomitant use of mEHT can significantly reduce the risk of shorter overall survival, compared to those receiving classical treatment options only. Moreover, all of the previous studies and the current pilot study also reported that the treatment is safe; adverse events may occur occasionally, and, in most cases, only grade I side effects occur.

Of the two cancers, cervical cancer is a bit more researched in terms of mEHT thanks to a randomized clinical trial conducted by Minnaar et al. [22,29]. They reported that chemoradiotherapy with concomitant mEHT can significantly reduce 2- and 3-year disease recurrence, result in a longer overall survival [22], and increase the complete metabolic resolution of all disease inside and outside the radiation field [30] compared to those patients receiving chemoradiotherapy only. Moreover, no increase in the frequency of late toxicities was reported between the treated and non-treated cohorts [22,29]. Similarly, both the overall response (e.g., complete response, partial response, etc.) and no-evidence-of-disease rates improved following the use of mEHT [23,25,31], but only a marginal difference was found in the case of disease-free survival [23]. It has to be mentioned though that pelvic lymph node metastases significantly affected disease-free survival in the study of Lee et al., and mEHT showed better results if the results were further analyzed in the subset of patients with pelvic lymph node metastases [23]. The overall survival rates, adverse event profiles, etc., were similar in our pilot as described in the earlier studies, and the very promising positive results of our meta-analysis suggest that, with a high probability, mEHT is a potent treatment modality to be used in everyday cervical cancer treatment. Therefore, further investigations into this cancer are urgently warranted.

Similar to cervical cancer, all previous publications have reported mainly positive results in ovarian cancer. In these, mEHT was described as being safe, with minimal treatment-related adverse events [24,26,32]. While an early case report presented a patient who was still in complete remission after four years [32], the other two available studies reported significantly shorter median overall (8 months in both studies) and progression-free (between 3 and 7 months) survival times [24,26]. With the median overall survival of 18 months, our own pilot study falls between these two main observations. The 1- and 2-year survival rates of ovarian cancer patients were 45% and 33%, based on the meta-analysis. Examining the survival results in detail, our pilot study’s results are more consistent with those of Kim et al. [24]. In general, it can be said about ovarian tumor studies that, compared to cervical cancer, the overall study designs are more limited by answering less questions, involving less and more heterogenous patients without comparator groups; therefore, the obtained results of this meta-analysis can only imply equivalence or benefit rather than making true conclusions about effectiveness. Based on the data available so far, it is likely that mEHT is effective in this patient population, but further exploratory studies are needed to truly answer this question.

In all the existing literature, including our own institutional results, the use of mEHT is a safe, non-invasive approach for the treatment of gynecological cancers. The most common side effects are a burning sensation at the treatment area, redness of the skin and/or grade I/II burn(s), which usually require no further medical care [4,5]. Moreover, its concomitant use with chemo(radio)therapy does not affect the adverse effect profile(s) of the other oncological treatment(s) [4,5,22,23,24,25,26,29,30,32]. Minnaar et al. [29] also examined the safety of mEHT in patients with comorbidities such as Acquired Immune Deficiency Syndrome (AIDS) and reported no increased toxicity or side effects in patients receiving the mEHT therapy, indicating the minimal effect it has on quality of life. Furthermore, Yoo et al. [26] investigated whether mEHT has any effect on quality of life. They have found that the physical well-being scores significantly decreased throughout the observational period; however, social, emotional, and functional well-being scores did not change at all [26]. In contrast, the various domains of psychosocial health for gynecological cancer patients/survivors who are on standard-of-care treatments are usually decreased [33].

Advanced cervical and ovarian cancers have poor survival rates. The 1- and 2-year survival rates of cervical [34] and ovarian [35] cancers are lower than 80% and around 60%, and approximately 70% and 50%, respectively. In our meta-analysis, we could show better survival rates for cervical cancer favoring the mEHT-treated cohort, while survival rates were somewhat worse for ovarian cancer. It has to be noted, however, that, in the study of Yoo et al., only such cervical patients were included into the study “who refused further chemotherapy” and “were not expected to benefit from” any additional systemic treatment [26]. Therefore, it is possible that, with further testing, the survival rate of mEHT-treated ovarian cancer patients will also improve. The improved survival rates of the mEHT patients, which might be an absolute risk reduction, can also appear due to the following theory. It was suggested before that the immunomodulatory effect of the treatment [36] can induce mechanisms that may also help turning immunologically cold tumors into hot ones [37]. If such mechanisms can be activated, mEHT combined with targeted and/or immunotherapies can open up new opportunities in the treatment of gynecological cancers. Since only a few questions regarding mEHT have been answered so far, the scope for future research is extremely broad.

### Limitations of the Study

Although, to the best of our knowledge, we are the first to analyze the results of mEHT studies in cervical and ovarian cancer in a meta-analysis, some limitations in the analysis should be mentioned. First and foremost, only a limited number of trials with sometimes very small sample sizes could be investigated, and only a single study was a randomized clinical trial. Additional limitations were raised due to the high heterogeneity of the patients, most patients having different and various treatments before the introduction of mEHT, some differences in the calculation of OS, the lack of appropriate control groups in the ovarian cancer studies, and the retrospective study design.

Similar limitations apply to our pilot study: the retrospective design, the small and heterogeneous patient population, the data originating from a single center only, and no further data that could be retrieved about the patients, such as details of irradiation therapy and detailed laboratory results before and after or throughout the mEHT treatments. Furthermore, no specific testing on subcutaneous adipose burns was performed.

## 5. Conclusions

The use of more advanced hyperthermia methods, including mEHT, shows promising results. In the current meta-analysis, it was found that the overall survival of gynecological cancer patients was better if concomitant mEHT was applied, especially for those with cervical cancer. To date, no study compared classical treatments with or without mEHT in ovarian cancer; therefore, further studies in this area are extremely needed. In conclusion, more (randomized) studies are required with large patient numbers. In cervical cancer, where a randomized trial is already available and the results of our meta-analysis also supported this, the treatment should be tested in real-life settings.

## Figures and Tables

**Table 1:** Relevant clinicopathological and treatment data of the six patients treated with modulated electro-hyperthermia (mEHT) at our institute. Survival time was calculated as the time elapsed between the initiation of the mEHT treatment and the date of death or the last follow-up (31-MAY-2025).

Patient ID	Age	Diagnosis	Tumor Location	Device	AMTD (W)	mEHT Count	Operation	Diagnosis-to-mEHT (Months)	Radioth. ^1^	Chemoth. ^1^	Pregnancies	Relevant Anamnesis	Familial History	Survival Time (Month)
Patient 1	57	2010	Cervix	EHY-2000+	133.89	18	Y	78	N	N	1	–	Negative	11 (D)
Patient 2	29	2016	Cervix	EHY-2000+	145.36	14	Y	24	Y	Y	0	Myoma	Paternal side: cervix, ovarian	3 (D)
Patient 3	88	2017	Ovarium	EHY-2030	140.2	50	N	5	N	N	1	–	Negative	13 (D)
Patient 4	48	2018	Ovarium	EHY-2030	120.31	16	Y	1	N	N	1	–	Negative	79 (A)
Patient 5	61	2016	Ovarium	EHY-2030	114.62	13	Y	51	N	Y	2	Cervical reconstr., curettage	Negative	64 (A)
Patient 6	36	2019	Ovarium	EHY-2000+	115.22	23	Y	17	N	Y	0	HSIL, ASCUS	Negative	18 (D)

A: alive; AMTD: average maximal treatment dose; ASCUS: atypical squamous cells of undetermined significance; D: died; HSIL: high-grade squamous intraepithelial lesion; N: no; Y: yes. ^1^ Radiotherapy or chemotherapy administered prior to the first mEHT treatment.

**Table 2:** Details of the selected studies investigating the effect of modulated electro-hyperthermia (mEHT) on gynecological cancer.

Author (Year)	Type of Study	Tumor	Cases (*n*)	Controls (*n*)	mEHT Device	Previous Adjuvant Therapy	Age (Median)
Lee 2017 [25]	RObs	Cervical	18	20	Oncotherm EHY2000+	Platinum based chemotherapy	52
Minnaar 2022 [22]	RCT	Cervical	100	102	Oncotherm EHY2000+	Radiochemotherapy	50
Lee 2023 [23]	RObs	Cervical	42	53	Oncotherm EHY2000+	Radiochemotherapy	59
Yoo 2019 [26]	PObs	Ovarian	19	–	Oncotherm EHY2000+	Chemotherapy	55
Kim 2021 [24]	PObs	Ovarian	12	–	Oncotherm EHY2000+	Chemotherapy: Paclitaxel or cisplatin	64

PObs: prospective observational study; RCT: randomized clinical trial; RObs: retrospective observational study.

## Data Availability

All data generated or analyzed in this study are included in this article and/or its tables and figures. Further enquiries can be directed to the corresponding author.

## Supplementary Materials

The following supporting information can be downloaded at: https://www.mdpi.com/article/10.3390/medsci14010105/s1,Table S1: PRISMA checklist.
